# Relationship between family function and anxiety among nurses during the COVID-19 pandemic: a mediating role of expressive suppression

**DOI:** 10.1186/s12912-024-02167-6

**Published:** 2024-07-29

**Authors:** Tianyi Bu, Chundi Peng, Jili Zhang, Bowen Wan, Lingbin Kong, Danni Xie, Boakye Kwame Owura, Jiawei Zhou, Xiaomeng Hu, Siyuan Ke, Kexin Qiao, Zhengxue Qiao, Yanjie Yang

**Affiliations:** Psychology and Health Management Center, Harbin Medical University, No.157, Baojian Road, Nangang District, Harbin, 150081 China

**Keywords:** Anxiety, COVID-19 pandemic, Expressive suppression, Family function, Nurses

## Abstract

**Background:**

The aims of the present study were to investigate the incidence of nurses who suffered anxiety during the COVID-19 pandemic and to explore how expressive suppression influences the relationship between family function and anxiety.

**Methods:**

This study used cross-sectional research and simple random sampling. A total of 300 questionnaires were distributed and 254 questionnaires were qualified after invalid questionnaires were proposed, thus a total of 254 female nurses from a tertiary hospital were included in this study. The measurement included General demographic questionnaires, the Self-Scale Anxiety, Scale (SAS), Emotion Regulation Strategies Questionnaire (ERQ), and Family Function Assessment (FAD). T-test, nonparametric Wilcoxon or Kruskal-Wallis test, χ2 test, Pearson or Spearman correlation analysis, multiple stepwise regression and bootstrap methods was performed to analyze the data.

**Results:**

In this study, 22.4% of the nurses exhibited anxiety symptoms, with 17.7% eliciting mild anxiety symptoms, 4.3% showing moderate anxiety symptoms and 0.4% with severe anxiety symptoms. Family function and expressive suppression were positively associated with anxiety severity. And family function influenced anxiety among nurses via direct and indirect (Expressive suppression -mediated) pathways.

**Conclusions:**

Expressive suppression partially mediated the influence of family function on anxiety symptom. To this end, nurse administrators should establish a robust mental health support system encompassing psychological counseling and emotional support groups. Furthermore, nurse administrators should consistently inquire about nurses’ family situations, encourage nurses to articulate their emotions and needs candidly, both within the domestic sphere and the workplace, while refraining from excessive self-repression.

## Introduction

Coronavirus disease 19 (COVID-19) was declared a pandemic within the first three months of its existence due to its rapid spread [1]. The COVID-19 outbreaks are extremely devastating and both developed and developing countries faced similar health challenges and medical stress. The sudden surge in the number of patients has led to overcrowding in hospitals and other health institutions [2]. In the context, the workload of the hospitasl has significantly increased, and nurses, as an important component of the hospitals, bear particularly important responsibilities and missions throughout the entire prevention and control process. However, facing high-intensity work pressure and infection risk fot a long period of time, the physical and mental health of the nurse population has been greatly challenged, especially the mental health problems are becoming more and more prominent, such as the frequent occurrence of anxiety and other psychological reactions [3].

Worldwide, it is estimated that 264 million individuals suffer from anxiety. But during medical outbreaks, nurses turn to have a greater rate of anxiety than the overall population [4, 5]. In different countries, the nursing profession is generally recognized as one of the high-pressure occupations, and nurses are more prone to anxiety compared to other healthcare workers [6, 7]. Studies have shown that the incidence of psychological anxiety and depression among healthcare workers under chronic stress is as high as 43.3%, which is more than twice as high as in other fields of work [8]. There are numerous reasons for this phenomenon, including, but not limited to, more demanding work, lack of personal protective equipment, fear of infection, and decreased social support [9, 10]. In addition, longer work hours, fewer days off, and absenteeism among nurses due to isolation for infections exacerbate anxiety levels [11]. The duration and severity of the COVID-19 pandemic further exacerbated anxiety levels in the nurse population [12].

Family function and emotional regulation play an important role as factors influencing an individual’s outlook on life and their mental health during the COVID-19 pandemic. The proper performance of basic family functions provides the necessary conditions for the healthy development of family members in physical, psychological and social aspects [13]. Family function is primarily concerned with how family members fulfill their various responsibilities and support each other [14]. It has been shown that good family function is associated with lower levels of anxiety, especially in studies conducted during the COVID-19 pandemic, which indicated that individuals with better family support were less likely to experience anxiety symptoms [15, 16].

Emotion Regulation is the process by which Individuals experience and express their emotions [17]. It mainly consists of cognitive reappraisal and expressive suppression, which have different effects on anxiety [17]. Cognitive reappraisal refers to an individual’s perception of the situation, assessment, and emotion management techniques employed in the face of stress [18]. Expressive suppression, on the other hand, is a maladaptive-focused emotion regulation strategy that alters the impact of a situation on emotions by inhibiting emotionally expressive behaviors [19]. Studies have shown that increased cognitive reappraisal skills can lead to a decrease in anxiety symptomatic events in lone patients, whereas increased expressive suppression may lead to increased anxiety in cancer patients [20, 21]. For the nurse population, anxiety that is not effectively controlled can affect nurses’ performance and job satisfaction in the long term, and even lead to frequent absenteeism and eventual resignation, which is not conducive to the development of healthcare [22]. The current nursing work model in China advocates for “patient-centered” and “high-quality nursing services”, emphasizing holistic services. Therefore, good emotional regulation ability plays a crucial role in the entire work process of nurses.

In summary, anxiety levels were generally high in the nurse population during the COVID-19 pandemic, and previous studies have confirmed that family functioning and emotion regulation play an important role in alleviating anxiety. Therefore, the aims of this study were to investigate the prevalence of anxiety in the nurse population during the COVID-19 pandemic, to analyze the relationship between family functioning and their anxiety status in the nurse population, and to explore the role of expressive inhibition in emotion regulation in the relationship between family functioning and anxiety in nurses. Through this study, it is expected that targeted suggestions and strategies can be provided to nurse managers during the COVID-19 pandemic in order to effectively maintain nurses’ mental health, improve work efficiency, enhance nursing quality, and thus promote the healthy development of nursing, and provide a more solid support and guarantee for the prevention and control of the pandemic.

## Methods and material

### Subjects

According to the latest statistics, of the more than 5 million nurses in China by the end of 2021, only 3% will be male. This figure highlights the fact that female nurses are in the absolute majority. Given that the focus of this study is on the overall status of the nurse population, and given the small number of male nurses in the hospitals surveyed, this study focuses on the female nurse population in order to ensure the representativeness and reliability of the data. In the selection of study subjects, we set clear inclusion and exclusion criteria. The inclusion criteria covered the following points: nurses who hold a nursing license, have served in the hospital for one year or more, have direct contact with infected persons, and must show a positive attitude and willingness to cooperate with this survey. The exclusion criteria explicitly excluded nurses who temporarily left their jobs during the survey period for various reasons.

In this survey, 300 questionnaires were proposed to be distributed. As of March 20, 2022, 282 questionnaires were collected, with a recovery rate of 94%. In order to ensure the quality and validity of the questionnaire data, we carried out a strict quality control and eliminated the questionnaires containing empty items, missing items and mutually exclusive questions. After this step, 254 qualified questionnaires were finally obtained, and the actual recovery rate was 90.07%, which meets the requirements of the sample size of this study.

### Methods

This study used a cross-sectional study and a simple random sampling method to investigate nurses working in a Grade A class three general hospital (It is a hospital that provides high-level specialized medical and health services and performs higher education and scientific research in several areas at a regional level or above.) in Harbin, Heilongjiang Province, in March 2022. Random sampling can ensure that the sample can reflect the overall characteristics of the nurse group in the top three hospital, minimize the bias, and increase the reliability of the results. Given the tension and complexity of hospital work during the COVID-19 pandemic, conducting a large-scale investigation could put an additional burden on daily hospital operations and nurses’ work. Therefore, with limited resources, we chose a sample size that is both representative and feasible. The specific steps are as follows:

Number all nurses in the hospital. Each nurse has a unique number. Random number generation: Use a computer random number generator to generate random numbers. According to the generated random numbers, nurses with corresponding numbers were selected as samples from the whole population. If the nurse corresponding to a random number cannot participate in the survey (such as leave, resignation, etc.), a random number is generated again for replacement.

### Data collection tools

#### -General Information Questionnaire

The general demographic questionnaire used in this study includes gender, age, registered residence location, marital status, maternity, education, income, family relationship, and relationship with parents, colleagues, friends, length of service, professional title, staffing, daily working hours, etc.

#### -Self-rating anxiety scale (SAS)

The SAS [23] includes 20 items and each item is scored on a four-point Likert scale. The raw score of the scale ranged from 20 to 80. In order to generate the index score, the raw score was multiplied by 1.25 and only the integer part was kept. The index score ranged from 25 to 100 with higher index scores on the SAS reflecting increasing levels of anxiety. When the total score of the questionnaire was less than 50, the participant had no anxiety symptoms; Mild anxiety was assessed when the total score was between 50 and 59.When the total score is between 60 and 69, the subject can be assessed to be in a moderate state of anxiety; When the total score of the respondents was ≥ 70, the respondents were assessed to be in a state of severe anxiety. The scale has good reliability and validity in nurse studies, with a Cronbach’s alpha coefficient of 0.915 in this study.

#### -Emotion regulation questionnaire (ERQ)

The Emotion Regulation Questionnaire (ERQ) contains two dimensions [19]: cognitive reappraisal (CR) and expressive suppression (ES). Cognitive reappraisal strategy refers to regulating one’s emotions through recognition, which contains a total of 6 items. The expression suppression strategy refers to controlling emotions by suppressing self-expression and contains a total of 4 items. The questionnaire uses a seven-level scoring method,. The score range of cognitive reappraisal strategy ranges from 6 to 42 points, and the score of expression suppression strategy ranges from 4 to 28 points, and the higher score of one dimension indicated which strategy the study subjects preferred to use. The questionnaire has good reliability and validity, and in this study the total questionnaire Cronbach’s alpha coefficient was 0.875, of which the Cronbach’s alpha coefficient for cognitive reappraisal was 0.762 and the Cronbach’s alpha coefficient for expressive inhibition was 0.816.

#### -Family Assessment device (FAD)

The Family Assessment Device (FAD), which contains 6 individual questionnaires and a Family Overall Functioning Rating Scale with 6 dimensions [24]: problem solving, communication, role, affective responsiveness, affective involvement, and behavior control. The scale contains a total of 60 items. Each item is a score of 4:. The total score obtained by adding all entries in each dimension is divided by the average number of questions in the dimension, which is the score of the dimension, with 1–2 points indicating good family function, 2.01-3 points indicating medium family function, and 3.01-4 points indicating poor family function. The scale had good reliability and validity in the nurses’ study, and the Cronbach’s alpha coefficient was 0.953 in this study.

### Statistical methods

The SPSS package (version 20.0 for Windows) was used for all data analyses. The t-test, ANOVA were used to analyze the differences in anxiety scores of nurses across demographic data. Correlation analysis and multiple stepwise regression analysis was used to assess the relationships among family function, expressive suppression, and anxiety. Mediation analysis was used to examine if expressive suppression (mediator variable) influenced the relationship between family function (independent variable) and anxiety (dependent variable), with working hours per day, co-worker relationships, the frequency of contacting with friends, and the frequency of doing exercise, treated as concomitant variables. All continuous variables were centralized to eliminate multi-collinearity before conducting the mediation analysis. The mediation effect was then tested by SPSS bootstrap analysis using 5,000 samples. All tests were two-sided, and statistical significance was set at *p* < 0.05.

## Results

### Analysis of general demographic data for nurses

A total of 254 participants were included in this study, of which, the age group is mainly concentrated in the 30–39 years old, accounting for about 70.5% (179 people) of the total number, 65.40% (166 people) are the only child, and married people account for about 29.10% (74 people) of the total proportion. The general demographics of the nurses are detailed in Table 1.

**Table 1:** The basic information of nurses in our research (*n* = 254)

Variable	Number of people	Constituent ratio (%)
Age		
< 30	38	15.00
30–39	179	70.50
40–49	24	9.40
> 49	13	5.10
**Whether is an only child**		
the only child	166	65.40
non-only child	88	34.60
**Marital status**		
Married	74	29.10
Unmarried	166	65.40
divorced	13	5.10
Widowed	1	0.40
**Maternal history**		
Infertility	92	36.20
produce one	132	52.00
produce two and above	30	11.80
**Seniority**		
less than one year	8	3.10
1 to 5 years	24	9.40
5 to 10 years	47	18.50
Above 10 years	175	69.00
**Department**		
internal medicine	79	31.10
Surgery	84	33.10
operating room	30	11.80
other departments	61	24.00
**Hours worked per day**		
within 8 h	85	33.50
8 ~ 12 h	139	54.70
more than 12 h	30	11.80
**Average monthly income**		
below 3000	14	5.50
3000 ~ 4500	94	37.00
4500 ~ 6000	92	36.20
6000 ~ 7500	34	13.40
7500 or more	20	7.90

### The score of anxiety 、ERQ and FAD scales among nurses

In this study, the nurses had an anxiety score of (42.45 ± 10.25), an expression suppression score of (16.69 ± 4.01) and a family function score of (25.10 ± 5.98).

### The incidence of anxiety among nurses during the pandemic

As shown in Table 22.4% of nurses had anxiety symptoms, among them,17.7% of nurses had mild anxiety, 4.3% of nurses got moderate anxiety, and 0.4% of nurses had severe anxiety.

**Table 2:** The incidence of anxiety among nurses

	Number	Percentage (%)
None	197	77.6
Mild anxiety	45	17.7
Moderate anxiety	11	4.3
Severe anxiety	1	0.4
Total	254	100

### Correlation analysis of ERQ、FAD and anxiety levels of nurses

Table 3 presents the results of the correlation analyses among family function, expressive suppression, and anxiety. Family function and expressive suppression were positively associated with anxiety severity, The correlation is significant at a significance level of 0.01 (*P* < 0.01).

**Table 3:** Correlation between family function, expressive suppression and anxiety

Scales	Family function	Expressive suppression
Family function	-	-
Expressive suppression	0.172^**^	-
Anxiety	0.473^**^	0.250^**^

^**^P < 0.01

### Relationship between family function and anxiety in nurses: the mediating role of expressive suppression

Hierarchical regression analysis (Table 4) revealed that family function was positively associated with anxiety (β = 0.443, *P* < 0.05) and expressive suppression was positively correlated with anxiety (β = 0.174, *P* < 0.05). These results suggested that the relationship between family function and anxiety was partially mediated by expressive suppression. The bootstrap tests (Table 5) indicated that this mediation effect was significant (95% CI: 0.0457–0.1204). The direct effect of family function on anxiety severity was also significant (95% CI: 0.0080–0.1083), indicating that family function influenced anxiety among nurses via direct and indirect (Expressive suppression -mediated) pathways.

**Table 4:** Mediation analysis of expressive suppression on the relationship between family function and anxiety

Variable	B	SE	T	*P*
Family function on anxiety	0.811	0.095	8.527	< 0.001
Family function on expressive suppression	0.116	0.042	2.775	0.006
Expressive suppression on anxiety(indirect)	0.444	0.141	3.138	0.002
Family function on anxiety(direct)	0.760	0.095	8.006	< 0.001

**Table 5:** Bootstrap results for the mediation analysis

Variable	Estimate	SE	LL95%CL	UL95%CL
Family function on anxiety(direct)	0.7203	0.0926	0.5379	0.9028
Expressive suppression on anxiety(indirect)	0.0519	0.0262	0.0080	0.1085

## Discussion

The COVID-19 pandemic has not just exposed the vulnerabilities in our health care delivery but has equally affected the mental health of the staffs involved in delivering the care. Study has shown that during the COVID-19 pandemic, nurses reported higher levels of anxiety when compared to other health workers [25]. The rapid spread of the epidemic has increased the demand for medical supplies and added to the work pressure of healthcare workers. Shortages of medical supplies, frequent and close contact with patients, and the resulting fear of being infected and infecting others exacerbate the psychological burden on nurses, which in turn leads to elevated levels of anxiety [9].

The prevalence of anxiety within our sample size was 22.4%. This was similar to the 26.9% prevalence of anxiety for nurses Huang reported in their study. Study also reported the prevalence of anxiety for doctors and administrative staffs to be 14.29% and 18.7% [25, 26] respectively being lower than the prevalence of anxiety within our study showing nurses had an increased risk of anxiety than other health workers. Due to the close connection between nurses and patients, they may be influenced by factors such as fear of infection, insufficient personal protective equipment, and high work intensity, which can lead to more psychological stress, then lead to higher levels of anxiety [27].

Family function was seen to have a positive outcome on anxiety of nurses indicating that nurses with good family support will have less anxiety while those with poor family support will have increased anxiety. The low rate of anxiety as compared to other studied may be due to the fact that most of the nurses are still in very good relationship with their parents indicating good family function [16]. During the epidemic, nurses, as the main part of hospital work, had a heavy workload and a high risk of infection. However, their family members were able to give nurses sufficient recognition and support in their work. Nurses were able to express their emotions and reduce work pressure and anxiety, allowing them to spare no effort in fighting the epidemic [28].

Family function appears to influence anxiety via two pathways, a direct pathway and an indirect pathway mediated by expressive suppression. This study demonstrated a direct association between expressive suppression, family function and anxiety. Nurses with a low level of family function were more likely to use expressive suppression, which in turn resulted in increased anxiety severity. In this study, the Emotion Regulation Questionnaire (ERQ) was used to investigate the regulatory strategies used by nurses in the face of difficult stress, mainly cognitive reappraisal and expressive suppression. The results showed that cognitive reappraisal strategy has a significant negative predictive effect on nurses’ anxiety while expressive suppression strategy has a significant positive predictive effect on nurses’ anxiety. Cognitive reappraisal could analyze the reasons of emotions before they occur and then make adjustments accordingly to improve those emotions [19]. Using cognitive reappraisal can effectively help an individual lower negative emotional experiences, which in turn present higher positive emotions leading to decreased anxiety [29]. Expressive suppression involves suppressing your internal emotions about the outside world [19], and as a maladaptive emotional coping strategy, often fails to reduce anxiety symptoms in nurses [30]. The results of this study further support this view. Habitual long-term suppression of thought and emotional expression is considered an important cause of depression and anxiety [31]. Having decreased expressive suppression over a period will lead to having lower anxiety while having an increase expressive suppression will lead to higher anxiety [21]. During the epidemic, the workload of nurses was heavy and the risk of infection increased. Nurses were under high pressure for a long time. If they were unable to vent their emotions and express their complaints in a timely manner for a period of time, but instead hid them in their hearts and pretended that everything was fine, there may be a situation where complaints are constantly hidden and anxiety increases [32]. In reality, strong family function can aid in the lowering of an individual’s expressive suppression which may lead to reducing anxiety. The findings of this study allow us to draw the conclusion that individuals will have low anxiety levels when families reduce the levels of expressive suppression.

In addition, the results of this study are generalizable and replicable. It is not only applicable to the group of nurses during an epidemic, but can equally be extended to other work settings and to a wider range of healthcare workers. Nurses always play a pivotal role in the healthcare system, and they face a heavy workload and great occupational stress regardless of whether an epidemic occurs or not. These stresses stem from multiple factors such as long hours of work commitment, complex interactions with patients, and dealing with medical emergencies. Therefore, the intrinsic link between nurses’ expression inhibition and anxiety revealed in this study is equally applicable in other groups of nurses in other work scenarios. Meanwhile, other professionals in the healthcare system, such as physicians and medical technicians, face similar work stress and emotional management challenges as nurses. Therefore, the findings of this study regarding the effects of family functioning on individuals’ expression of repression and anxiety can be generalized to these healthcare workers as well.

### Limitations and Future directions

There are a few limitations to the study. First, this study is a cross-sectional study conducted at a single point in time, so it is not possible to determine the temporal order between the variables, and a cross-sectional study can only reveal the associations between the variables and cannot exclude other potential factors that may affect the results. In this study, there may be other factors related to the new crown epidemic and the anxiety status of nurses, such as individual personality, work pressure, and social support, which may have an impact on the results, but the cross-sectional study was unable to directly consider the role of these factors, and therefore causality could not be determined. Secondly, with the intense and complex nature of hospital work during the New Crown epidemic, conducting a large-scale survey may place an additional burden on the daily operations of the hospital and the work of the nurses. Therefore, the sample size was relatively small. Last, the anxiety rate of the population before COVID-19 was not known making it hard fully link the prevalence of anxiety to COVID-19 related issues or personal stressors.

Effective interventions should be implemented to reduce the anxiety of nurses. The influence a family’s support has on decrease the anxiety levels of nurses should not be undermined. Nurses should learn to talk, no longer suppress negative emotions in their hearts, and be good at seeking comfort and help from family and friends. Moreover, Medical and health regulatory authorities and hospitals should pay attention to the mental health level of nurses, mental health education courses and training should be carried out, so as to achieve timely intervention and treatment. In short, to alleviate nurses’ anxiety, we should flexibly use a variety of emotional regulation strategies, supplemented by regular screening and physical exercise, to help nurses establish a healthy emotional experience and emotional expression system and stay away from anxiety. The government and hospital managements should be open to giving nurses allowances and incentives for the extra work they provide.

## Conclusions

In summary, the present study suggests the higher anxiety level of nurses during the COVID-19 pandemic may be due to the frequent use of emotional regulation strategies of expressive suppression, which exacerbated the influence of family functions on anxiety. Therefore, the formation of more adaptive and targeted interventions for family function and expressive suppression may reduce nurses’ anxiety, thereby maintaining and improving nurses’ mental health.

## Data Availability

The datasets used and/or analyzed during the current study are available from the corresponding author on reasonable request.
